# Effect of Acteoside as a Cell Protector to Produce a Cloned Dog

**DOI:** 10.1371/journal.pone.0159330

**Published:** 2016-07-18

**Authors:** Ji Hye Lee, Ju Lan Chun, Keun Jung Kim, Eun Young Kim, Dong-hee Kim, Bo Myeong Lee, Kil Woo Han, Kang-Sun Park, Kyung-Bon Lee, Min Kyu Kim

**Affiliations:** 1 Division of Animal & Dairy Science, College of Agriculture and Life Science, Chungnam National University, Daejeon, Republic of Korea; 2 Department of Biology Education, College of Education, Chonnam National University, Gwangju, Republic of Korea; Peiking university third hospital, CHINA

## Abstract

Somatic cell nuclear transfer (SCNT) is a well-known laboratory technique. The principle of the SCNT involves the reprogramming a somatic nucleus by injecting a somatic cell into a recipient oocyte whose nucleus has been removed. Therefore, the nucleus donor cells are considered as a crucial factor in SCNT. Cell cycle synchronization of nucleus donor cells at G0/G1 stage can be induced by contact inhibition or serum starvation. In this study, acteoside, a phenylpropanoid glycoside compound, was investigated to determine whether it is applicable for inducing cell cycle synchronization, cytoprotection, and improving SCNT efficiency in canine fetal fibroblasts. Primary canine fetal fibroblasts were treated with acteoside (10, 30, 50 μM) for various time periods (24, 48 and 72 hours). Cell cycle synchronization at G0/G1 stage did not differ significantly with the method of induction: acteoside treatment, contact inhibition or serum starvation. However, of these three treatments, serum starvation resulted in significantly increased level of reactive oxygen species (ROS) (99.5 ± 0.3%) and apoptosis. The results also revealed that acteoside reduced ROS and apoptosis processes including necrosis in canine fetal fibroblasts, and improved the cell survival. Canine fetal fibroblasts treated with acteoside were successfully arrested at the G0/G1 stage. Moreover, the reconstructed embryos using nucleus donor cells treated with acteoside produced a healthy cloned dog, but not the embryos produced using nucleus donor cells subjected to contact inhibition. In conclusion, acteoside induced cell cycle synchronization of nucleus donor cells would be an alternative method to improve the efficiency of canine SCNT because of its cytoprotective effects.

## Introduction

SCNT technique has been used to produce genetically superior or manipulated animals for agricultural purposes and as biomedical resources. With the increasing need for animal models of disease, rescuing animals in danger of extinction, stem cells for regenerative medicine, organ transplantation, etc., the interest in cloning of animals using SCNT has been increasing in recent times [1–5]. Even though the production of cloned animals has been successful, the efficiency of SCNT is still very low [6]. The causes of the low SCNT embryo developmental competence include many factors which are related to the quality of recipient oocytes, quality of the nucleus donor cells and the condition of the surrogate mother [7–10].

Reactive oxygen species (ROS) and apoptosis have been known to be involved in female reproductive processes including oocyte development *in vivo* and *in vitro* [11–14]. ROS interrupt the embryo development by inducing cytoplasmic condensation, DNA damage and apoptosis in embryos. Supporting studies have reported that reducing ROS improves SCNT embryo development competence during *in vitro* culture [15–17]. Apoptosis is a physiological process that occurs spontaneously during normal preimplantation embryo development to maintain cellular homeostasis by removing DNA damaged/malfunctioning cells. ROS causes DNA fragmentation and increases apoptotic blastomeres that disturb embryo development. The event of ROS and apoptosis is observed more often after SCNT than after *in vitro* fertilization [18, 19]. Many studies reported that SCNT embryo development competence is rescued by reducing the levels of ROS and apoptosis by using antioxidants such as selenium [20], insulin-transferrin-selenium [17, 21], melatonin [22] and glutathione [23].

Previous studies reported that the use of donor cells arrested at the G0/G1 stage improved the efficiency of SCNT [7, 9, 24–28]. The chromatin in the donor cells at G0/G1 stage has been considered the most effective for SCNT embryo development competency. To synchronize the donor cell cycle, contact inhibition, serum starvation, and chemical treatments have been frequently used [7, 25, 29, 30]. However serum starvation reduced cell survival and increased DNA fragmentation, which leads to apoptosis [31]. Chemical inhibition is another strategy to arrest the cell cycle at the G0/G1 stage using inhibitors such as roscovitine [29, 32], dimethyl sulfoxide (DMSO) [33], and cycloheximide (CHX) [34]. The efficiency of the method used for synchronization is affected by the types and species of the donor cells. For optimal donor cell synchronization, various aspects of the methods used need to be carefully evaluated.

Acteoside (also known as verbascoside) [2-(3, 4-dihydroxyphenylethyl)-1-O-a-L-rhamnopyranosyl-(1 */* 3)-b-D-(4-O-caffeyl)-glucopyranoside] is isolated from the violet flowers of *Syringa vulgaris*. Plants containing acteoside have been known to have anti-microbial and antifungal activities [35–37], and prevent inflammation. Indeed, acteoside exhibits various biological activities *in vitro* and *in vivo* such as anti-oxidative activity, anti-apoptotic activity, cytotoxicity against various tumor cells and cell cycle synchronization at the G0/G1 stage as a cyclin-dependent kinase (CDK) inhibitor [38]. For example *Lee et al*. [38] reported that acteoside treatment can inhibit the proliferation of human promyelocytic HL-60 leukemia cells by inducing the cell cycle arrest at the G0/G1 stage. However, the effect of acteoside-treated donor cells on the efficiency of canine SCNT has yet to be studied.

In this study, the effects of acteoside on the cell cycle synchronization, ROS and apoptosis on canine fetal fibroblasts were investigated. The analysis of cell cycle synchronization, ROS and apoptosis was performed using fluorescence activated cell sorting (FACS). To demonstrate the efficiency of the donor cells treated with the acteoside, SCNT embryos were cultured and transferred into a recipient dog, and successfully produced a healthy cloned dog.

## Materials and Methods

### Ethics statement

In this study, 44 female mongrel dogs (aged 1–6 years) were used as oocyte donors and embryo transfer surrogates, and a female beagle (aged 2 years) was used for establishment of fetal fibroblast cell lines. All animal experiments were approved by Institutional Animal Care and Use Committee (IACUC) of Chungnam National University (Approval Number: CNU-00487), and performed according to “The Guide for the Care and Use of Laboratory Animals” published by IACUC of Chungnam National University. Dogs were raised indoors in separate cages with a temperature and ventilation control system. Dogs were fed commercial diet once daily and provided water *ad libitum*. In all the animal experiments, dogs were anaesthetized with 6 mg/kg ketamine and xylazine initially, and maintained in anaesthetized condition with 2% isoflurane. At the end of the surgery, antibiotics (Procain Penicillin G, Benzathine Penicillin G, and Dihydrostreptomycin sulfate) were administrated to prevent any infection. After surgery, the dogs were transferred to their own individual cages with fresh water, monitored, and provided special care from the veterinarian in charge, so that necessary treatments could be administered if the animals needed it.

### Chemicals

All chemicals were purchased from Sigma-Aldrich Chemical Co. (St. Louis, MO, USA) unless otherwise stated. Acteoside was obtained from Chengdu Biopurify Phytochemicals Ltd. in China.

### Isolation of canine fetal fibroblasts

Canine fetal fibroblasts were obtained from 28-day old fetuses. Briefly, a female beagle was artificially inseminated with sperm of a male beagle. After 28 days, pregnancy in the female beagle was confirmed and 5 fetuses were obtained by celiohysterectomy. Fetuses were washed thrice in phosphate-buffered saline (PBS; Gibco, Thermo Fisher Scientific Inc., Waltham, Massachusetts, USA). The head, internal organs and limbs of the fetuses were removed with a surgical blade and the remaining body parts were minced in PBS. Pieces of fetuses were washed twice by centrifugation at 400 × *g* for 5 min and cultured in Dulbecco’s Modified Eagle’s Medium (DMEM; Gibco) supplemented with 1% penicillin-streptomycin (product No. P4458) and 20% fetal bovine serum (FBS; Gibco) at 39°C in a humidified atmosphere of 5% CO_2_ and 95% air. When cells reached confluence after 5–7 days, they were treated with 0.25% trypsin-EDTA (Gibco) for 2 min and harvested. Two canine fetal fibroblast cell lines were established from two individual fetuses. The cells were frozen in 10% DMSO in FBS and stored in liquid nitrogen at -196°C until they were used.

### Cell treatment

The frozen canine fibroblasts were thawed and cultured until 80% confluency. Cells were harvested and passaged 4 to 7 times for subsequent experiments. Cells were seeded at a concentration of 10^6^/mL in 60 mm culture dishes. The cells were cultured in (1) DMEM supplemented with 10% FBS and 1% penicillin-streptomycin for five days (contact inhibition), (2) DMEM supplemented with 0.5% FBS and 1% penicillin-streptomycin for five days (serum starvation), or (3) DMEM supplemented with 10% FBS and 1% penicillin-streptomycin containing acteoside at 10 μM, 30 μM, or 50 μM for 24 h, 48 h, and 72 h (acteoside treatment).

### Cell cycle analysis by flow cytometry

For analysis of the cell cycle stages, cells were harvested using 0.25% trypsin-EDTA and washed 3 times with PBS by centrifugation. After the last wash, the supernatants were discarded and the cells resuspended in 0.3 mL PBS. For fixation, 0.7 mL cold absolute ethanol was added drop-wise to the cell suspension, mixed by gentle vortexing, and stored overnight at 4°C. The fixed cells were centrifuged and washed twice with cold PBS. The cells were resuspended in 0.25 mL PBS supplemented with 5 μL of 10 mg/mL RNase and incubated for 1 h at 37°C. Then, 10 μL of 1 mg/mL propidium iodide (PI) was added. Cell cycle was analyzed by quantitation of DNA content in cell populations at G0/G1 (2n), S (between 2n and 4n), and G2/M (4n) phases using a FACS Calibur flow cytometer (Becton Dickinson, San Jose, CA, USA).

### ROS detection by flow cytometry

ROS in live cells was assessed using an Image-iT^™^ Live Green Reactive Oxygen Species Detection Kit (Molecular Probes, Eugene, OR, USA). Briefly, cells were washed in PBS after removing culture medium, and 2 mL of the 100 μM *tert*-butyl hydroperoxide (TBHP) working solution was added. After incubation at 37°C for 1 h, TBHP working solution was removed, and the cells were gently washed 3 times in warm Dulbecco’s phosphate-buffered saline (DPBS; Gibco). Cells were then incubated in 25 μM 5-(and-6)-carboxy-2,7–dichlorodihydrofluorescein diacetate (carboxy-H2DCFDA) working solution at 37°C for 30 min in the dark. When oxidized, carboxy-H2DCFDA emits green fluorescence, facilitating the assessment of ROS by flow cytometry. TBHP, which is an inducer of ROS production, was used a positive control. The nuclei of the cells were stained with Hoechst 33342. After washing 3 times with PBS, cells were harvested by trypsinization, suspended in PBS, and used for detecting ROS by using a FACS Calibur flow cytometer (Becton Dickinson, San Jose, CA, USA). The rate of ROS formation was calculated by dividing the number of ROS positive cells with the total cell number, and converting the values into percentages.

### Apoptosis analysis by flow cytometry

Apoptosis analysis was performed using a Vybran^®^ Apoptosis Assay Kit #2 (Molecular Probes, Eugene, OR, USA). Briefly, the cells were harvested by trypsinization, and washed 2 times with cold PBS. Thereafter, 100 μL of 1× annexin-binding buffer, 5 μL of Alexa Fluor 488 annexin V, and 1 μL of PI were added to the cells. After incubation for 15 min at room temperature, 400 μL of 1× annexin-binding buffer was added and the cell suspension was gently mixed. Annexin V, which is a Ca^2+^ -dependent phospholipid-binding protein has a high affinity for phosphatidylserine (PS). In live cells, PS is located on the inner cytoplasmic membrane, and translocated to the outer cytoplasmic membrane in apoptotic cells. Apoptotic cells are distinguished by Annexin V labeled with Alexa Fluor 488 binding to PS on the outer membrane. In addition, PI, which is a nucleic acid binding dye, stains necrotic cells with red fluorescence. Therefore, apoptotic cells with green fluorescence, necrotic cells with red fluorescence, and live cells with little or no fluorescence in the cells were analyzed using a FACS Calibur flow cytometer (Becton Dickinson, San Jose, CA, USA). The rates were calculated by dividing the cell numbers of live, necrotic, or apoptotic cells with the total cell number, and converting the calculated values into percentages.

### Collection of *in vivo* matured oocytes

To detect ovulation in each dog, serum progesterone (P4) concentration was measured using a DSL-3900ACTIVE progesterone Coated-tube Radioimmunoassay Kit (Diagnostic Systems Laboratories, Inv., TX, USA). As previously described [39, 40], the day when P4 concentration exceeded 4 ng/mL was considered as the day of ovulation. At 72 h after ovulation, oocytes were retrieved by laparotomy using aseptic surgical procedures. The fimbria of the oviduct was approached through the bursal slit and cannulated using an inverted flanged bulb steel needle (18 gauge, 7.5 cm). A 24 gauge intravenous (IV) catheter (Angiocath^™^ Plus, Becton Dickison Korea Inc., Seoul, Korea) was inserted into the isthmus of the oviduct near the uterotubal junction, and medium 199 (Gibco) supplemented with 1% penicillin-streptomycin and 5% FBS was introduced into the oviduct through the IV catheter. Oocytes were immediately transported to the laboratory. A total of 316 oocytes were collected from 31 dogs.

### Somatic cell nuclear transfer and embryo transfer

SCNT was performed as previously described [39]. Briefly, to remove cumulus cells from *in vivo* matured oocytes, cumulus-oocyte-complexes (COC) were repetitively pipetted in 0.1% hyaluronidase. The first polar body and metaphase-II chromosomes were removed by aspiration. Enucleation was confirmed by staining with Hoechst 33342 (bisbenzimide). Canine fetal fibroblasts were treated with 30 μM acteoside for 48 h and then harvested with 0.25% trypsin-EDTA. A single cell, which has smooth cell membrane, was transferred into the perivitelline space of each enucleated oocyte. The couplets were equilibrated in fusion medium, which is 0.26 M mannitol medium containing 0.1 mM HEPES, 0.5 mM MgSO_4_ and 0.05% BSA, and fused by two pulses of direct current (69–75 V for 15 μs) from an Electro-Cell Fusion apparatus (NEPA gene Co., Chiba, Japan). These couplets were incubated in medium 199 supplemented with 10% FBS for 1 h 30 min. The fused SCNT embryos were checked and chemical activation was induced by incubating them in 10 μM calcium ionophore (product No. C7522, Sigma) for 4 min. SCNT embryos were washed and then placed in modified synthetic oviductal fluid medium (mSOF) [41] containing 1.9 mM 6-dimethylaminopurine (6-DMAP) for 3 h 30 min. The engineered embryos were surgically transferred into the oviducts of oestrus-synchronized surrogates within 4 h after SCNT. Pregnancy was confirmed by ultrasonography at 26 days (at the earliest) after embryo transfer.

### *In vitro* culture of cloned embryos

After SCNT, cloned embryos were cultured in 40 μL microdrops of modified synthetic oviductal fluid medium (mSOF) (10 cloned embryos per microdrop) overlaid with mineral oil at 38.5°C in 5% CO_2_ and 95% air. SCNT embryo development was observed from day 2 to day 8.

### Microsatellite and mitochondrial DNA analysis of a cloned dog

Total DNA was extracted from the nuclear donor cells, and the skin of the oocyte donor dogs, surrogate dogs and the cloned dog by the G-spin^™^ Genomic DNA Extraction Kit (iNtRON BIOTECHNOLOGY, Seongnam, Korea) according to the manufacturer’s guidelines, and then was eluted in 100 μL of TE buffer (10 mM Tris-HCl, 1 mM EDTA, pH 8.0). The amount of DNA was measured by the BioSpec-nano Spectrophotometer (Shimadzu Corp., Japan).

For microsatellite genotyping, the following 7 specific genes were selected: PEZ1, PEZ3, PEZ8, PEZ12, FH2010, FH2054, and FH2079. PCR was performed with initial denaturation for 10 min at 95°C, 20 cycles comprised of denaturation for 30 sec at 95°C, 30 sec of annealing at 58°C (-0.1°C/cycle), 1 min of extension at 72°C, and 15 more cycles comprised of denaturation for 30 sec at 95°C, 30 sec of annealing at 56°C, 1 min of extension at 72°C, and final extension at 72°C for 10 min.

For mitochondrial DNA sequencing, using the complete nucleotide sequences of canine mtDNA (GenBank accession no.: U96639), the following oligonucleotide primers were synthesized: Cytochrome b region (L14,252 –L14,631) F: ACTCATTCATTGACCTCCCAGCG, R: AGTTCCGATATAAGGGATGGCAGAG; Cytochrome c oxidase subunit II (L7,054—L7,465) F: ATGGCGTACCCATTTCAACT, R: GGATGGTTATTTCTATTGG; 16S rRNA (L2,033 –L2,472) F: GCAAAGGTAGCATAATCAT, R: AGGACTTTAATCGTTGAAC; D-loop region (L15,622 –L16,030) F: CATAGGACATATTAACTCAATC, R: AAGTCCAGCTACAAGTTATTTG [42]. The PCR cycles for this analysis included: 95°C initial denaturation for 5 min, 5 cycles of denaturation for 30 sec at 95°C, 40 sec of annealing at 60°C (-1°C/cycle), 1 min of extension at 72°C, and the next 30 cycles of denaturation for 30 sec at 95°C, 40 sec of annealing at 55°C, 1 min of extension at 72°C, and final extension at 72°C for 10 min. PCR fragments from the various experiments were analyzed using an ABI Prism 3100 Genetic Analyzer (Applied Biosystems Inc., USA). Sequence assembly, multiple alignments, and alignment trimming for control regions were performed with the BioEdit software (v.7.0.5.3).

### Statistical analysis

Data were analyzed using IBM SPSS statistics (SPSS Inc., Chicago, IL USA) by one-way ANOVA and Tukey’s honestly significant difference test. Differences were considered statistically significant when *P* value was less than 0.05.

## Results

### Effect of acteoside on the cell cycle synchronization

The effect of acteoside on cell cycle synchronization was investigated using fluorescence activated cell sorting (FACS) (Table 1). Canine fetal fibroblasts were treated with various concentrations of acteoside (10, 30, 50 μM) for different durations (24, 48, 72 h), and the cells were separated into G0/G1 stage, S stage and G2/M stage by FACS. The results of FACS were compared with those of the cells synchronized by serum starvation and contact inhibition. Serum starvation resulted in the highest rate of cell cycle synchronization (88.2%) at the G0/G1 stage compared to those of the contact inhibition (84.6%) and acteoside treatment (80.8–84.5%). Acteoside treatment for 24 h induced G0/G1 cell cycle synchronization at the lowest efficiency (80.8, 81.1, and 82.0%). Acteoside was most effective in inducing G0/G1 cell cycle synchronization at 30 μM concentration and treatment duration of 48 h (84.5%). However, there was no significant difference among concentrations and durations of acteoside treatment in the induction of G1/G0 cell cycle synchronization. Overall, acteoside treatment showed no significant difference compared to serum starvation and contact inhibition in terms of G0/G1 cell cycle synchronization. There was also no difference in cell cycle synchronization at S stage among the three groups. In addition, the proportion of cells arrested at the G2/M stage was similar to that of cells arrested at G0/G1 stage.

**Table 1:** Effect of contact inhibition, serum starvation, and acteoside treatment on cell cycle synchronization in canine fetal fibroblasts.

			Cell cycle stage, % (mean ± SE)		
Group			G0/G1 stage	S stage	G2/M stage
Contact inhibition			84.6 ± 1.2^ab^	2.6 ± 0.4	12.4 ± 2.5^AB^
Serum starvation			88.2 ± 0.6^a^	2.0 ± 0.3	7.8 ± 2.8^A^
		24 h	80.8 ± 1.4^b^	3.4 ± 0.3	15.2 ± 2.4^B^
	10 μM	48 h	82.4 ± 1.0^ab^	3.2 ± 0.5	14.0 ± 2.4^AB^
		72 h	84.2 ± 0.9^ab^	2.5 ± 0.3	12.8 ± 2.0^AB^
		24 h	81.1 ± 1.6^b^	3.0 ± 0.3	15.3 ± 2.7^B^
Acteoside	30 μM	48 h	84.5 ± 1.2^ab^	2.5 ± 0.4	12.7 ± 2.6^AB^
		72 h	84.2 ± 1.1^ab^	2.4 ± 0.3	13.0 ± 2.6^AB^
		24 h	82.0 ± 1.7^b^	3.0 ± 0.3	14.5 ± 3.0^B^
	50 μM	48 h	82.5 ± 1.6^ab^	2.8 ± 0.5	14.4 ± 3.3^B^
		72 h	84.2 ± 1.0^ab^	2.7 ± 0.3	12.9 ± 2.5^AB^

This experiment was repeated five times independently.

Values with different superscripts are statistically significant (*P*<0.05).

### Effect of acteoside on ROS and apoptosis

**Fig 1:**
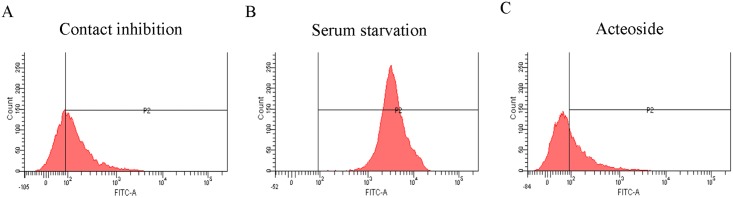
ROS levels in canine fetal fibroblasts. Cell cycle synchronization by (A) contact inhibition, (B) serum starvation and (C) 30 μM acteoside treatment for 48 h. Histograms show levels of ROS detected in canine fetal fibroblasts.

The effect of acteoside on ROS was determined by FACS using carboxy-H2DCFDA, an oxidative stress indicator that is activated in cells when esterases remove acetate groups between cells (Fig 1). ROS was detected at lower levels in acteoside treated cells than in cells synchronized by serum starvation or contact inhibition. The level of ROS was significantly lower in the acteoside treatment group (42.8%) than in the contact inhibition and serum starvation groups (54.3 and 99.5% respectively) (Table 2).

**Table 2:** Quantification of ROS

Groups	Rate (mean ± SE)
Contact inhibition	54.3 ± 4.2^a^
Serum starvation	99.5 ± 0.3^b^
Acteoside^†^	42.8 ± 1.8^c^

This experiment was repeated four times independently.

^†^ 30 μM acteoside for 48 hours.

Values with different superscripts differ significantly (*P*<0.05).

**Fig 2:**
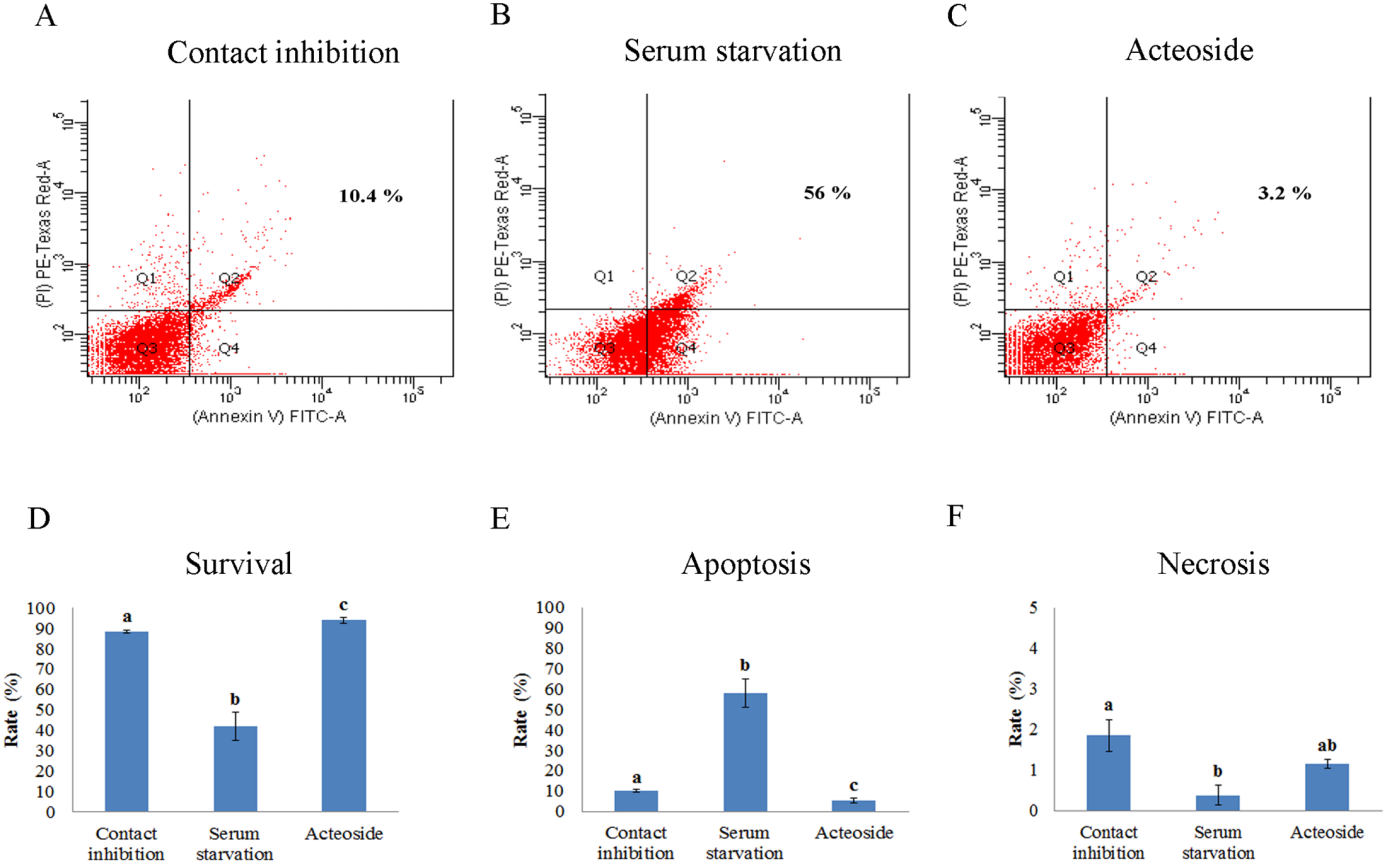
Rates of cell survival, apoptosis and necrosis of canine fetal fibroblasts. Apoptotic cells in the (A) contact inhibition, (B) serum starvation and (C) 30 μM acteoside treatment for 48 h group were determined by FACS; Quantitative analysis of the FACS results of (D) cell survival, (E) apoptosis, and (F) necrosis. This experiment was repeated four times independently. (*P*<0.05)

To understand the effect of acteoside on cell survival and death, apoptosis was investigated in cells after induction of cell cycle synchronization. Survival rate of acteoside-treated cells (94.8 ± 1.2%) was significantly higher than both contact inhibition and serum starvation groups (87.3 ± 0.5%) (Fig 2D). The rates of apoptosis in the serum starvation group (52.1 ± 5.3%) were significantly higher than those in the contact inhibition and acteoside treatment groups (Fig 2E). Apoptosis observed in the contact inhibition group was 10.6%, which was also significantly higher than the acteoside treatment group (4.2%) (Fig 2E). In addition, there was less necrosis found in acteoside treated cells compared to that in the contact inhibition group (Fig 2F).

### Effect of acteoside on *in vitro* development of canine nuclear transfer embryos

To investigate the effect of acteoside in SCNT embryo development, nuclear donor cells were transferred into canine enucleated oocytes after cell cycle synchronization by acteoside. As shown in Table 3, there were no significant differences in early embryo development from the nuclear donor cells with different cell cycle synchronization methods of either contact inhibition or acteoside treatment. However, only the canine SCNT embryos engineered by using the nuclear donor cells synchronized at G0/G1 stage with acteoside developed beyond the 10-cell embryo stage.

**Table 3:** *In vitro* development of canine nuclear transfer embryos.

	No. of embryos	Fused	Cleaved	4-cell	8-cell	>10-cell
		(%)	(%)	(%)	(%)	(%)
Contact inhibition	46	22	17	15	7	0
		(47.5)	(35.2)	(30.5)	(13.7)	
Acteoside^†^	58	35	26	22	11	3
		(60.8)	(45.1)	(38.4)	(16.9)	(5.8)

This experiment was repeated five times independently.

^†^ 30 μM acteoside treatment for 48 h.

### Production of a cloned dog using cells cultured with acteoside

**Fig 3:**
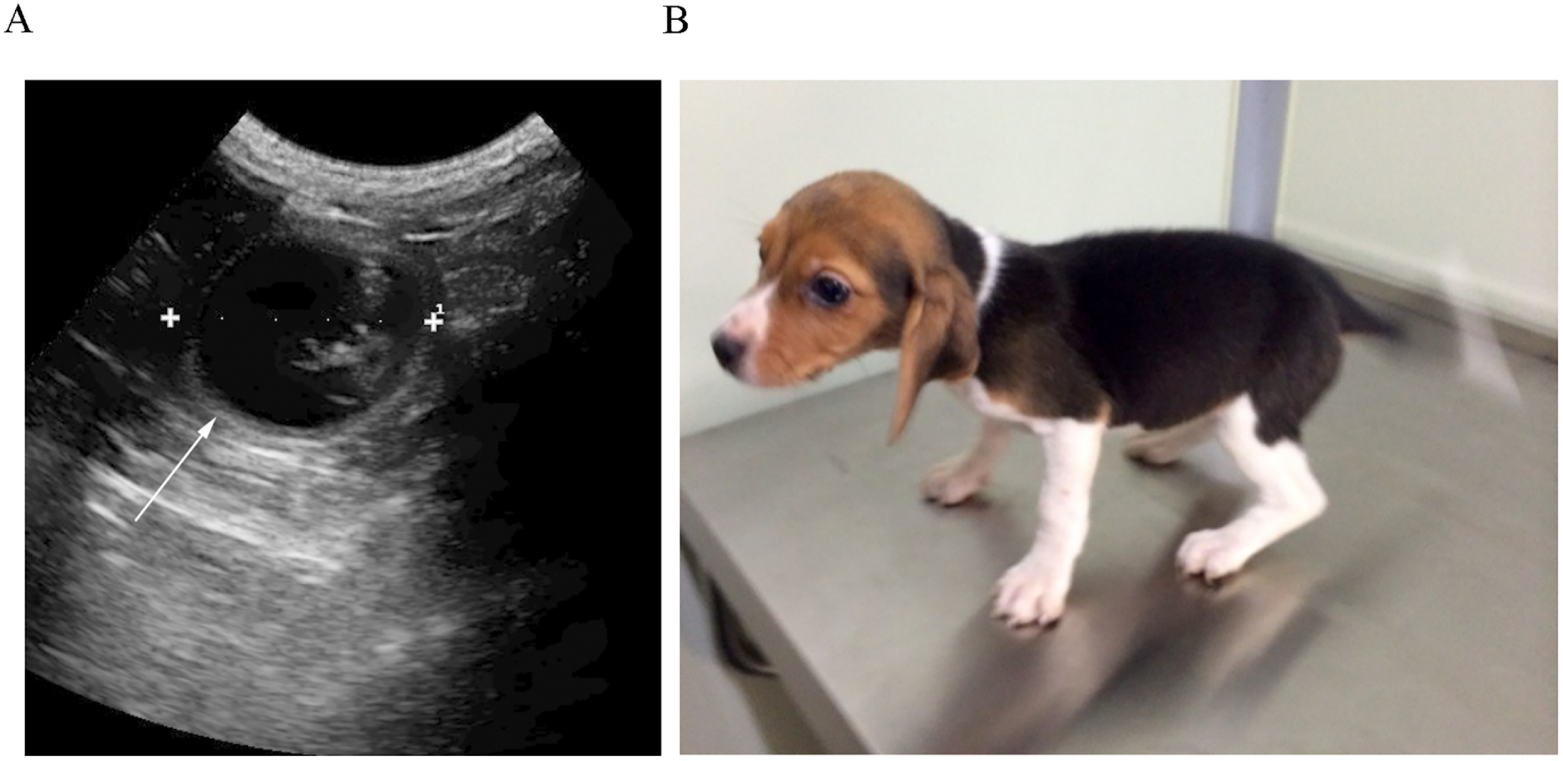
A cloned dog: (A) Ultrasonogram of the fetus in the fetal vesicle at 32 days after embryo transfer; (B) The cloned beagle at 2 months of age.

Fifty-seven cloned canine embryos produced using contact inhibited donor cells were surgically transferred into the oviducts of 6 recipients. None of the 6 recipients produced offspring or even became pregnant. Thirty-eight cloned canine embryos produced from acteoside-treated donor cells were also surgically transferred into the oviducts of 3 recipients. Pregnancy was confirmed in one of the three surrogate mothers, who eventually gave birth, by cesarean section, to a healthy puppy weighing 354 g (Table 4 and Fig 3). To determine the origin of the genome, microsatellite amplification was performed with genes of the cloned dog. Microsatellites are short tandem repeated sequences used in genome screens for tracing the heredity and linkage analysis in families. Seven microsatellite markers were used to identify the origin of the genome in the cloned dog (Table 5). The cloned dog and donor cells used to clone the dog possessed the same microsatellite markers, whereas the oocyte donor and surrogate shared only partial alleles (Table 5). In maternal ancestry test, the cloned dog and the recipient oocyte had identical mitochondrial DNA (mtDNA) sequences, showing that the mtDNA of the cloned dog was derived from the oocyte donor (Table 6).

**Table 4:** Production of a cloned dog by SCNT.

Groups	No. of transferred embryos	No. of recipients	No. of pregnancy	No. of cloned offspring
			(pregnancies/recipients, %)	(births/transferred Embryos, %)
Contact inhibition	57	6	0	0
Acteoside	38	3	1	1
			(33.3)	(2.6)

This experiment was repeated three times with acteoside group and six times with contact inhibition group independently.

**Table 5:** Microsatellite analysis of the cloned dog.

Marker	PEZ 1	PEZ 3	PEZ 8	PEZ12	FH 2010	FH 2054	FH 2079
Oocyte donor	119/127	120/120	236/244	299/303	227/231	171/171	276/280
Cloned dog	115/119	120/120	232/236	273/281	231/239	163/167	276/280
Nucleus donor cell	115/119	120/120	232/236	273/281	231/239	163/167	276/280
Surrogate	115/123	120/120	240/240	269/277	235/235	143/175	276/276

**Table 6:** mtDNA sequences of the cloned dog.

	Nucleotide position*																
	2207	2254	7084	7089	7096	7100	7109	7257	7272	7369	7389	7412	7460	14354	14408	14450	14492
Oocyte donor	T	A	G	C	A	A	A	T	T	C	-	C	A	G	G	C	T
Cloned dog	T	A	G	C	A	A	A	T	T	C	-	C	A	G	G	C	T
Nucleus donor cell	C	G	A	T	G	G	A	C	C	T	G	T	G	A	C	T	C
Surrogate	T	A	A	C	A	A	G	T	T	C	G	C	A	A	C	T	C

*GenBank Accession Number: U96639.

## Discussion

Synchronization of the donor cell cycle is an important factor to improve SCNT efficiency [43]. The rate of SCNT embryo development and the efficiency of cloning animal productions are improved with the use of donor cells at the G0/G1 stage relative to the use of donor cells at G2/M stage [24–28], although it has been reported that donor cells arrested at the G2/M stage of cell cycle can produce viable cloned piglets [44]. The cell cycle stage of the nucleus donor cells plays a crucial role in the reprogramming events that follow SCNT. Nucleus donor cells arrested at G0/G1 stage efficiently initiate the first DNA synthesis after SCNT [28, 29, 45]. To induce cell cycle synchronization, various chemical inhibitors including acteoside have been used to achieve cell cycle synchronization [46, 47]. As a CDK inhibitor, acteoside is often used to bring about cell cycle synchronization at the G0/G1 stage. Lee *et al*. reported that acteoside hindered the cell cycle progression beyond the G1 phase, thus preventing leukemia cell proliferation. In addition, the level of CDK was reduced but the levels of CDK inhibitors was significantly increased [38].

The present study compared the effects of acteoside to the other two common cell synchronization methods to investigate the effect of cell synchronization on the efficiency of SCNT. Canine fetal fibroblasts were treated with various concentrations of acteoside, serum starvation, and contact inhibition; the percentage of cells at the G0/G1 stage in the three treatment groups was compared. Serum starvation was found to be the most effective method for cell cycle synchronization at the G0/G1 stage, and there was no significant difference between acteoside and contact inhibition. However, serum starvation induced a significantly higher level of ROS. Previous studies reported that the increase of ROS damages cell membranes and induces apoptosis thereby diminishing the efficiency of embryo development. Moreover, ROS increases DNA fragmentation that induces the cell block and delays the embryo development in humans and pigs [48–51]. Acteoside treatment showed no difference in synchronizing cell cycle at G0/G1 stage compared to contact inhibition. However, acteoside induced significantly less ROS activity compared to the other two cell cycle synchronization methods. In addition, acteoside treatment induced significantly less apoptosis and necrosis than contact inhibition and serum starvation. The result is also congruent with the previous studies that showed the occurrence of more apoptotic events after cell cycle synchronization with serum starvation than with contact inhibition [32, 52]. Concurrent with the reduction in the rate of apoptosis, the acteoside treatment group also showed higher cell survival than the contact inhibition group. Serum starvation resulted in massive cell death compared to both acteoside treatment and contact inhibition.

Nucleus donor cell cycle synchronization at G0/G1 stage is a crucial step in successful SCNT embryo and ultimately in the production of cloned animals. ROS has been regarded as one of main causes of cell death and apoptosis during embryo development. In this study, acteoside was investigated to determine whether it would be a useful alternative method for inducing G0/G1 stage cell-cycle synchronization in canine fetal fibroblasts as nuclear donor cells. Induction of cell cycle synchronization by acteoside treatment of nuclear donor cells reduced ROS and apoptosis, which contributed to the improvement of *in vitro* development of SCNT embryos. Embryos cloned using acteoside-treated donor cells were transferred into surrogate mother dogs and one healthy cloned dog was produced successfully, which did not happen with embryos from the contact inhibition group.

In conclusion, this study demonstrated that acteoside, which is a CDK inhibitor, induces successful cell cycle synchronization of the canine fibroblasts at the G0/G1 stage for use as nuclear donor cells, and also protects them from apoptosis by reducing oxidative stress. The cytoprotective effect of acteoside, combined with the ability for cell cycle synchronization, contributed to improve the *in vitro* developmental competence of SCNT embryos. Therefore, acteoside would be an effective reagent to enhance the cloning efficiency to produce cloned animals.
